# Characterizing the Ovarian Cytogenetic Dynamics of Sichuan Bream (*Sinibrama taeniatus*) During Vitellogenesis at a Single-Cell Resolution

**DOI:** 10.3390/ijms26052265

**Published:** 2025-03-04

**Authors:** Zhe Zhao, Shixia Huang, Qilin Feng, Li Peng, Qiang Zhao, Zhijian Wang

**Affiliations:** 1 Integrative Science Center of Germplasm Creation in Western China (Chongqing) Science City, Southwest University, Chongqing 401329, China; zhaozhe023979@163.com (Z.Z.); 13708175889@163.com (S.H.); 13216335633@163.com (Q.F.); luckzhao0823@163.com (Q.Z.); 2 Key Laboratory of Freshwater Fish Reproduction and Development (Ministry of Education), School of Life Sciences, Southwest University, Chongqing 400715, China; pengli0811@163.com

**Keywords:** single-nucleus RNA sequencing, ovary, vitellogenesis, *Sinibrama taeniatus*

## Abstract

Vitellogenesis in fish represents a critical phase of oogenesis, significantly influencing the nutritional provisioning for oocyte maturation and subsequent offspring development. However, research on the physiological mechanisms governing vitellogenesis at the single-cell level remains limited. In this study, we performed single-nucleus RNA sequencing (snRNA-seq) on the ovaries of Sichuan bream (*Sinibrama taeniatus*). We first identified six distinct cell types (germ cells, follicular cells, immune cells, stromal cells, endothelial cells, and epithelial cells) in the ovaries based on typical functional marker genes. Subsequently, we reconstructed the developmental trajectory of germ cells using pseudotime analysis, which describes the transcriptional dynamics of germ cells at various developmental stages. Additionally, we identified transcription factors (TFs) specific to germ cells that exhibit high activity at each developmental stage. Furthermore, we analyzed the genetic functional heterogeneity of germ cells and follicular cells at different developmental stages to elucidate their contributions to vitellogenesis. Finally, cell interaction analysis revealed that germ cells communicate with somatic cells or with each other via multiple receptors and ligands to regulate growth, development, and yolk acquisition. These findings enhance our understanding of the physiological mechanisms underlying vitellogenesis in fish, providing a theoretical foundation for regulating ovarian development in farmed fish.

## 1. Introduction

Ovarian development in fish is a dynamic process characterized by distinct stages and continuous progression. Vitellogenesis is the most critical stage of ovarian development, characterized by phase-specific changes in ovarian cellular morphology, structure, and gene expression. Additionally, it involves the coordinated allocation of nutrient and energy reserves within the organism [1]. Vitellogenesis is defined as the process of producing and transporting yolk material, which accumulates in the oocyte and determines the nutritional level for oocyte maturation and subsequent offspring development [2]. Numerous scholars have extensively investigated the synthesis and transport of substances [3], energy metabolism [4], and gene expression [5,6] during fish vitellogenesis. The ovarian tissue is structurally complex and highly heterogeneous, containing various cell types. Throughout development, the ovaries undergo continuous structural and functional changes, with the gene expression profiles of each cell type experiencing dramatic alterations [7]. Moreover, the intricate interplay between germ and somatic cells plays a crucial role in ovarian development [8]. Consequently, understanding the dynamic changes in gene expression among key cell types in the ovary during vitellogenesis, as well as the interactions between these cells, is of significant importance.

In recent decades, RNA sequencing technology has rapidly advanced, becoming an invaluable tool for investigating gene expression and elucidating gene function, thereby enhancing researchers’ understanding of intricate biological processes. Compared to traditional bulk RNA sequencing technologies, single-cell RNA sequencing (scRNA-seq) can comprehensively reveal transcriptomic information within individual cells, enabling the identification of different cell types and molecular regulatory mechanisms specific to each cell type, which is a significant advantage in analyzing complex tissues that are constantly changing [9,10]. Currently, scRNA-seq has been gradually applied to reveal the cytogenetic dynamics of ovarian tissues in several mammalian species, including humans [11], monkeys [12], mice [13], yaks [14], and pigs [15]. In the realm of fish, scRNA-seq has provided unprecedented resolution in characterizing cellular heterogeneity and functional diversity, enhancing our understanding of oogenesis, gonadal development, and reproductive regulation. Recent studies on species such as Asian seabass (*Lates calcarifer*) [16], *Cynoglossus semilaevis* [17], and zebrafish (*Danio rerio*) [18] have successfully identified various ovarian cell types, including germ cells (such as oogonia and oocytes) and multiple somatic cells (such as follicular cells, stromal cells, and various immune cells). These studies have also elucidated key gene regulatory networks, such as TGF-β, Wnt, and Notch signaling pathways, as well as steroidogenesis and estrogen signaling pathways, which highlight their roles in ovarian development and maturation. Furthermore, based on single-cell transcriptomics, gene knockout experiments in zebrafish have demonstrated the roles of *foxl2l* and *wnt9b* in oocyte development and sex determination, respectively [18].

However, current studies remain less comprehensive compared to the vast diversity of fish species and their potential physiological variations. Additionally, there is a significant lack of single-cell transcriptome studies focusing on ovaries during the mid-to-late stages of development, particularly during the vitellogenesis phase. This gap arises because conventional scRNA-seq sample preparation platforms are unable to capture cells with diameters exceeding 40 μm [19]. In contrast, oocyte diameters typically surpass this threshold during the mid-to-late developmental stages, presenting a unique challenge for ovarian single-cell sequencing technologies. As single-cell sequencing technology continues to advance, single-nucleus RNA sequencing (snRNA-seq) has emerged as an alternative method for assessing a cell’s transcriptome through the isolation of nuclei. This technique has been utilized in studies of ovaries in species such as mice [20], goats [21], and Nile tilapia [22]. Furthermore, comparisons between nuclear and whole-cell transcriptomes have demonstrated a high degree of concordance between the two methods in identifying cell types and metabolic markers [23,24]. Consequently, snRNA-seq offers an effective approach for characterizing the dynamics of gene expression in cell types from previously inaccessible tissues.

Sichuan bream (*Sinibrama taeniatus*) is a small, economically valuable species of the Cyprinidae family, endemic to the upper reaches of the Yangtze River. According to previous field surveys [25], the average body length and weight of adult Sichuan bream are approximately 82.34 mm and 10.58 g, respectively, with a maximum recorded age of 4 years based on scale analysis. This species exhibits two annual breeding seasons, occurring in spring and autumn, and is characterized by a batch-spawning, asynchronous reproductive strategy. In our previous study, we delineated the boundaries of distinct stages of Sichuan bream oogenesis and further explored the patterns of material transport and energy metabolism during vitellogenesis [4]. Additionally, we identified members of the Vitellogenin (*vtg*) gene family in Sichuan bream and conducted a comprehensive analysis of their structural characteristics and expression patterns [26]. However, further investigation is warranted to elucidate the underlying physiological mechanisms regulating vitellogenesis. In this study, we performed snRNA-seq on ovarian tissues from the pre-vitellogenesis and vitellogenesis stages to construct a single-cell transcriptomic atlas of the Sichuan bream ovary and analyze the stage-specific genetic characteristics of germ cell development. Furthermore, we focused on comparing the transcriptional dynamics of germ cells and follicular cells between the pre-vitellogenesis and vitellogenesis stages. We also constructed a cell communication network to explore the interaction patterns among ovarian cells during vitellogenesis. This study is the first to examine the physiological mechanisms of fish vitellogenesis at single-cell resolution, advancing our understanding of reproductive biology and providing a foundation for regulating ovarian development in farmed fish.

## 2. Results

### 2.1. Single-Cell Transcriptome Atlas of the Sichuan Bream Ovary

**Figure 1:**
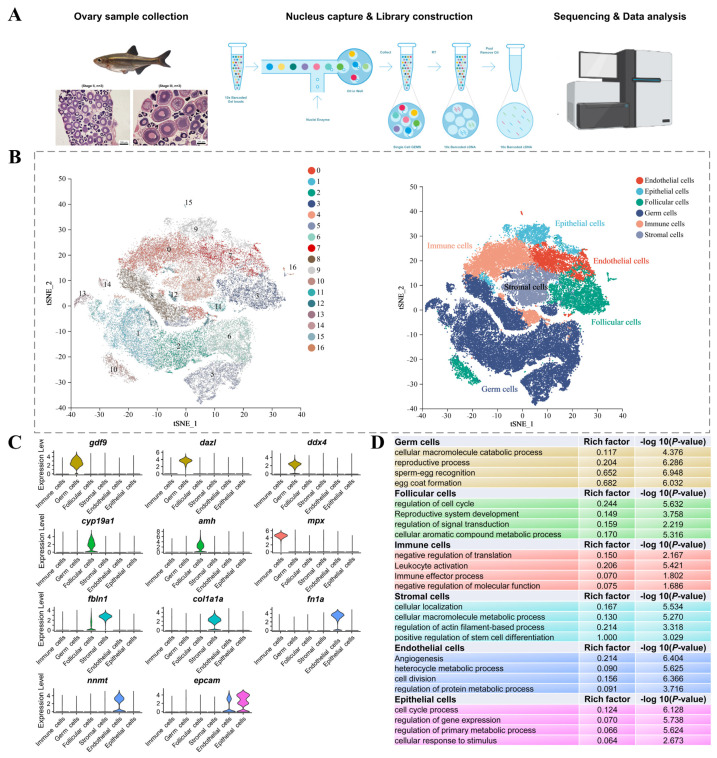
Identification of cell types in Sichuan bream ovaries using snRNA−Seq. (**A**) Flowchart of single−cell nuclear sequencing. The ovarian histological images are from our recent report [4]. (**B**) t−SNE analysis demonstrating the clustering (**left panel**) and identification (**right panel**) of ovarian cells. (**C**) Specific expression of marker genes in different cell types. (**D**) GO functional enrichment of DEGs among different cell types.

To comprehensively obtain single-cell transcriptome information related to vitellogenesis in Sichuan bream, fresh ovarian tissues were collected from female juvenile fish at various stages of ovarian development, up to pre-vitellogenesis (stage II, *n* = 3) and during vitellogenesis (stage III, *n* = 3), for the preparation of single-nuclei suspensions. Utilizing the 10× Genomics platform, six ovarian snRNA-seq libraries were constructed and sequenced (Figure 1A). After quality control and the removal of mitochondrial RNA, red blood cells, doublets, and ambient RNA, a total of 55,889 high-quality transcriptional profiles of cells were obtained for downstream analysis, comprising 29,477 germ cells and 26,412 somatic cells (Table 1).

All cells were clustered into 17 clusters using the “FindClusters” function, and the results were visualized using the non-linear dimensionality reduction algorithm t-distributed stochastic neighbor embedding (t-SNE) (Figure 1B). The outcomes were subsequently annotated into six distinct cell types based on multiple typical specific marker genes as follows: germ cells (*gdf9*, *dazl*, and *ddx4*) [27,28,29], follicular cells (*amh* and *cyp19a1*) [30,31], immune cells (*mpx*) [32], stromal cells (*fbln1* and *col1a1a*) [14,18], endothelial cells (*nnmt* and *fn1a*) [33], and epithelial cells (*epcam*) [34] (Figure 1C). Figure 1D demonstrates the specific expression of each marker gene within its corresponding cell type, thereby confirming the accuracy of our classification.

Additionally, GO enrichment analysis was conducted for DEGs that were significantly upregulated in each cell type to characterize their functions (Figure 1D). The terms “reproductive process”, “sperm-egg recognition”, and “egg coat formation” were significantly enriched in germ cells. In follicular cells, there was significant enrichment of “reproductive system development”, “regulation of signal transduction” and “cellular aromatic compound metabolic process”. DEGs in immune cells mainly participated in “leukocyte activation” and “immune effector process”. For stromal cells, DEGs were involved in “cell localization” and “regulation of actin filament-based process”. Additionally, endothelial cells showed significant enrichment in terms of “angiogenesis” and “cell division”. Epithelial cells exhibited significant enrichment in terms of “regulation of gene expression” and “cell cycle processes”. These findings show that the GO enrichment results of upregulated DEGs in each cell type align with the established physiological functions of the respective cells.

### 2.2. Dynamics of Gene Expression in Germ Cells During Development

**Figure 2:**
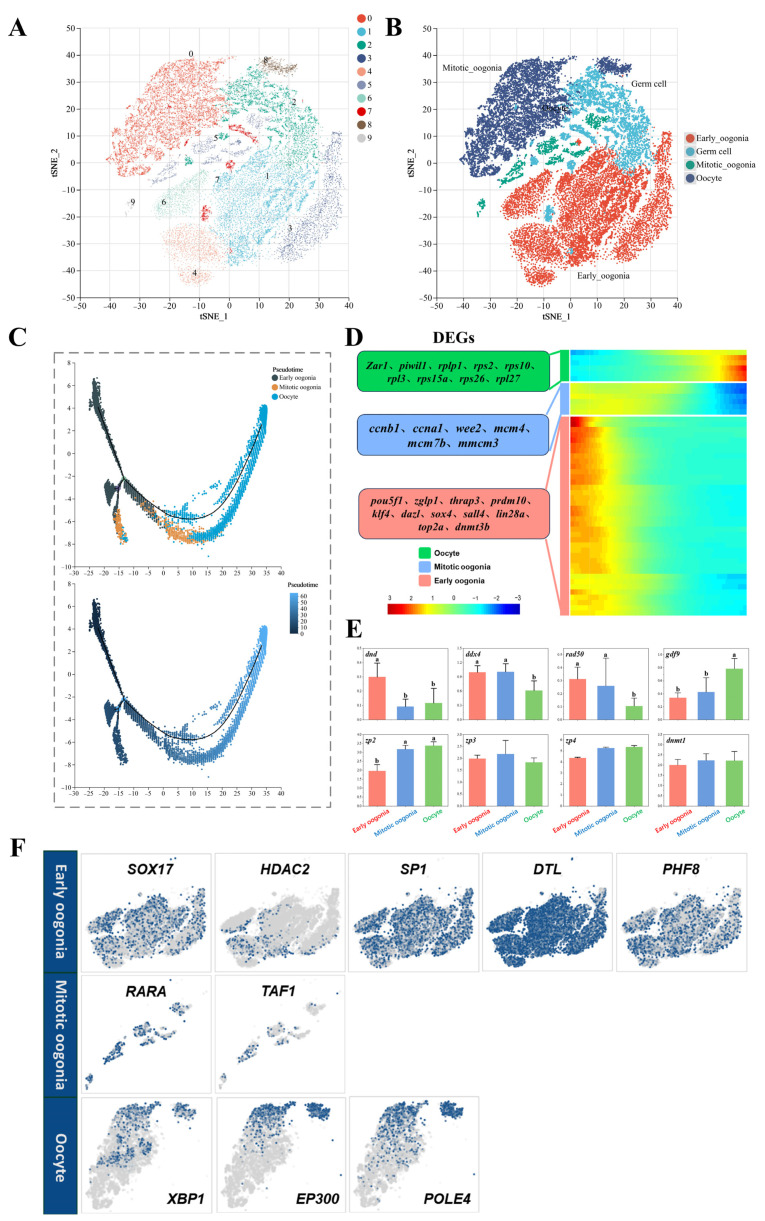
Dynamics of gene expression in germ cells during development. (**A**) Clustering of germ cell subtypes. (**B**) Identification of germ cell subtypes. (**C**) Developmental pseudotime trajectory of germ cell subtypes; different colors in the upper side represent different cell types, and the pseudotime trajectory in the lower side indicates the degree of cell differentiation, with darker colors indicating less differentiation. (**D**) Gene expression heatmap over pseudotime. (**E**) The expression levels of marker genes in three subtypes of germ cells; ^a,b^ mean values with unlike letters were significantly different (*p* ˂ 0.05). (**F**) Transcription factors of different germ cell subtypes.

To investigate the genetic dynamics of germ cells during development, we conducted a re-cluster analysis of germ cell types, resulting in the identification of nine subclusters at a resolution of 0.2 (Figure 2A). Clusters 1, 3, 4, and 6 were annotated as early oogonia (*pou5f1*) [35,36]; clusters 5 and 9 as mitotic oogonia (*rad50*) [37,38]; and clusters 0 and 8 as oocytes (*gdf9*) [11,39], based on the specific genes expressed by germ cells at distinct developmental stages. Since clusters 2 and 7 lack characteristic genes for specific stages, we still define them as germ cells (Figure 2B). Monocle2 was employed to construct the developmental trajectory of germ cells. The results of this analysis aligned with established developmental patterns, demonstrating that early oogonia, mitotic oogonia, and oocytes form a continuous pseudotime trajectory in accordance with their developmental order (Figure 2C). We observed that potential functional marker genes are specifically expressed at different developmental stages along pseudotime processes (Figure 2D). Among these, several typical early germ cell marker genes, including *pou5f1*, *zglp1*, *thrap3*, *prdm10*, *klf4*, *dazl*, *sox4*, *sall4*, *lin28a*, *top2a*, and *dnmt3b*, were found to be enriched in the early oogonia types. Cell division markers (*wee2*, *mcm4*, *mcm7b*, and *mmcm3*) and cell cycle regulators (*ccnb1* and *ccna1*) were enriched in mitotic oogonia. In addition to the typical germ cell marker genes *zar1* and *piwil1*, a significant number of ribosomal protein-related genes, including *rplp1*, *rps2*, *rps10*, *rpl3*, *rps15a*, *rps26*, and *rpl27*, were also observed to be enriched in the oocyte types. Furthermore, we compared the differential expression of several typical germline marker genes across three developmental stages (Figure 2E). The expression of *dnd* was highest during the early oogonia stage and was significantly downregulated in the subsequent two developmental stages (*p* < 0.05). The expressions of *ddx4* and *rad50* were upregulated in oogonia but significantly downregulated in oocytes (*p* < 0.05). The expression levels of *gdf9* and *zp2* increased with developmental time (*p* < 0.05), while the expression levels of *zp3*, *zp4*, and *dnmt1* remained consistently high at each detection stage (*p* > 0.05).

The developmental dynamics of germ cells are regulated by stage-specific transcription factors (TFs). Following the aforementioned analysis, SCENIC analysis was utilized to identify multiple TFs present in significantly upregulated gene sets across different subtypes of germ cells. As illustrated in Figure 2F, the TFs predominantly expressed in early oogonia include *sox17*, *hdac2*, *sp1*, *dtl*, and *phf8*. In mitotic oogonia, the identified TFs are *rara* and *taf1*. In oocytes, the detected TFs are *xbp1*, *ep300*, and *pole4*. These TFs may play a crucial role in maintaining the stage-specific functional characteristics of cells or in determining cell fate.

### 2.3. Effect of Vitellogenesis on the Gene Expression Profile of the Follicle Cell Population

**Figure 3:**
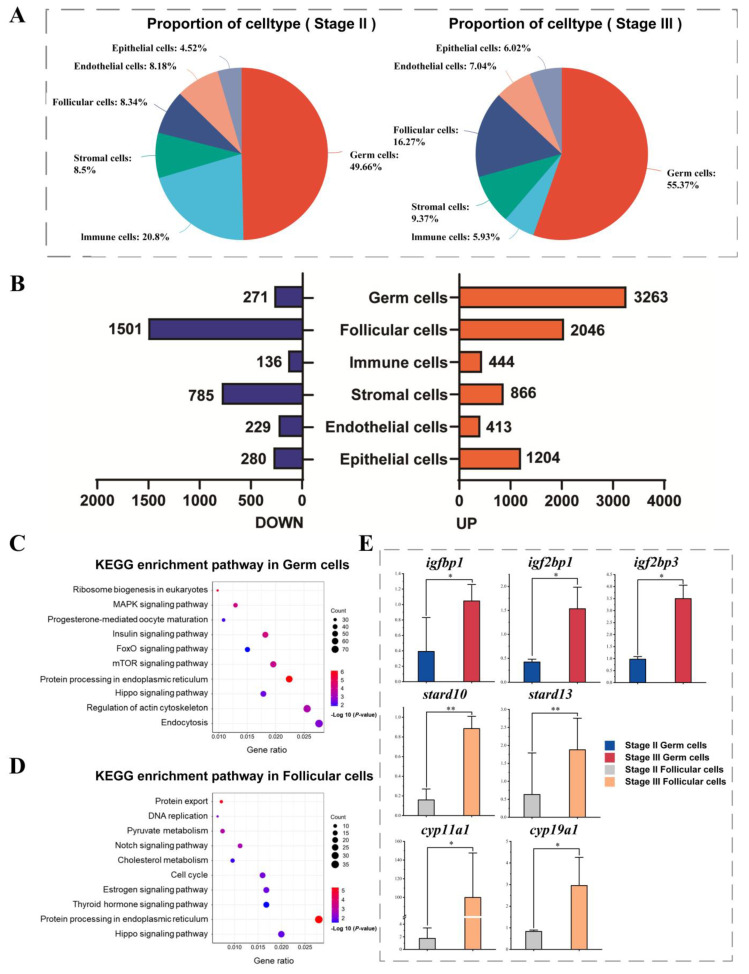
Effect of vitellogenesis on the gene expression profile of the follicle cell population. (**A**) Pie chart of proportional numbers of each cell type. (**B**) Numbers of up- and downregulated DEGs in the different cell types. (**C**) KEGG enrichment analysis of upregulated DEGs in germ cells during stage III. (**D**) KEGG enrichment analysis of upregulated DEGs in follicular cells during stage III. (**E**) The expression of DEGs at different developmental stages. * Mean values were significantly different (*p* ˂ 0.05); ** Mean values were highly significantly different (*p* ˂ 0.01).

To investigate the cellular changes associated with vitellogenesis, we compared cellular data from stage II and stage III ovarian samples (Supplementary Figure S1). In terms of quantitative proportions (Figure 3A), upon entering stage III, the abundance of germ cells and follicular cells, which are two critical components of ovarian follicles, increased significantly, with their proportions rising by approximately 5.7% and 7.9%, respectively. In contrast, the proportions of epithelial cells, endothelial cells, and stromal cells exhibited less variation in stage III compared to stage II. Furthermore, the number of immune cells significantly decreased by approximately 14.9% upon entering stage III. These findings indicate that vitellogenesis significantly remodels the cellular architecture of the Sichuan bream ovary. Additionally, DEGs from different cell types at the two developmental stages were compared (Figure 3B). Notably, germ cells exhibited the most significant changes, with 3263 upregulated and 271 downregulated DEGs, followed by follicular cells, which showed 2046 upregulated and 1501 downregulated DEGs (Figure 3B).

The follicle, consisting of germ cells and their surrounding follicular cells, represents the most fundamental functional unit in ovarian tissue. To elucidate the regulatory pathways of follicles during vitellogenesis, KEGG enrichment analysis was conducted on DEGs specifically upregulated in germ cells and follicular cells during stage III. In germ cells, the upregulated DEGs are mainly enriched in several functional modules, including the “insulin signaling pathway”, which pertains to endocrine control. Notably, insulin-like growth factor binding proteins *igfbp1*, *igf2bp1*, and *igf2bp3* are significantly upregulated in stage III. Additionally, DEGs are enriched in pathways related to “progesterone-mediated oocyte maturation”, “MAPK signaling pathway”, “mTOR signaling pathway”, “Hippo signaling pathway”, and “FoxO signaling pathway”, all of which are associated with molecular signaling. Furthermore, pathways linked to protein synthesis, such as “protein processing in endoplasmic reticulum” and “ribosome biogenesis in eukaryotes”, were also found to be enriched (Figure 3C). The DEGs specifically upregulated by follicular cells in stage III primarily involve “protein export” and “pyruvate metabolism”, which are related to energy metabolism and substance transport; “DNA replication” and “cell cycle”, which pertain to cell division and proliferation; “Notch signaling pathway” and “Hippo signaling pathway”, which are involved in molecular signaling transduction; as well as the “estrogen signaling pathway”, “thyroid hormone signaling pathway”, and “cholesterol metabolism”, which are related to hormone metabolism (Figure 3D). Among these DEGs, StAR-related lipid transfer proteins *stard10 and stard13*, along with cytochrome P450 family members *cyp11a1* and *cyp19a1*, were significantly upregulated in stage III.

### 2.4. Cell Communication Analysis Based on Ligand–Receptor Pairs

**Figure 4:**
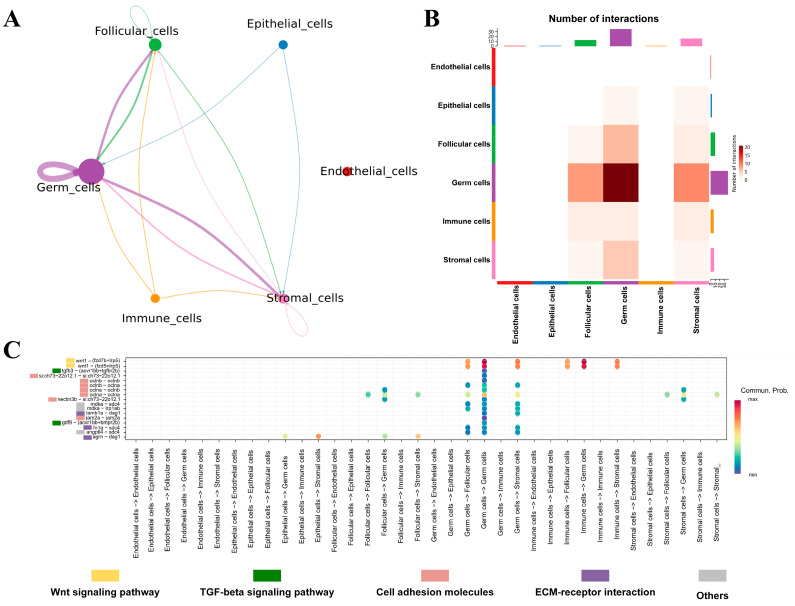
Cellular communication during vitellogenesis. (**A**) Intercellular communication network. (**B**) Intensity heatmap of intercellular interactions. (**C**) Pathways involved in ligand–receptor pairs.

The development of germ cells is tightly regulated by the surrounding somatic cells. To investigate the complex interactions between ovarian cells during vitellogenesis, CellChat was employed to establish an intercellular communication network based on the expression levels of ligand–receptor pairs (Figure 4A). The results showed that germ cells primarily regulate follicular cells and stromal cells while also being influenced by follicular cells, stromal cells, epithelial cells, immune cells, and themselves. Meanwhile, the heatmap constructed based on the number of ligand–receptor pairs illustrated the strength of the regulatory relationship between cell types (Figure 4B). The germ cell group exhibited high-intensity internal communication and extensive interconnections with other cell types. As depicted in Figure 4C, germ cells are predominantly regulated by somatic cells through receptors such as *dag1*, *oclna*, *aclnb*, *sdc4*, *bmpr2*, and *lrp5*. Conversely, germ cells primarily exert influence on other cell types or regulate themselves by recognizing corresponding receptors through ligands including *jam2a*, *fn1a*, *nectin3b*, *lamb1a*, *oclna*, *oclnb*, *gdf9*, and *wnt1*. Further enrichment analysis of the ligand–receptor pairs revealed that during the vitellogenesis stage, ovarian cell communication is mediated by various pathways, including “cell adhesion molecules”, the “TGF-β signaling pathway”, the “Wnt signaling pathway”, and extracellular matrix (ECM) components, resulting in a wide range of interactions.

## 3. Discussion

### 3.1. Cell Composition of the Sichuan Bream Ovary

The development of single-cell transcriptome sequencing has provided unprecedented insights into intercellular gene regulation in ovarian tissue [40,41]. Identifying the cellular composition of the target tissue and constructing a cell atlas are fundamental components of single-cell studies [42]. Through single-cell sequencing of a substantial number of samples, it has been revealed that the human ovary comprises six predominant cell types: oocytes, granulosa cells, immune cells, endothelial cells, perivascular cells, and stromal cells [43]. Additionally, a scRNA-seq investigation indicated that the ovarian cell types in mice exhibited a high degree of concordance with those observed in adult humans, encompassing germ cells, granulosa cells, immune cells, endothelial cells, and stromal cells [13]. In the realm of single-cell studies on fish ovaries, it has been found that the ovaries of the Chinese tongue sole (*Cynoglossus semilaevis*) consist mainly of germ cells, follicle cells, endothelial cells, macrophages, T cells, and erythrocytes [17]. Furthermore, germ cells, follicle cells, theca cells, stromal cells, neutrophils, macrophages, NK cells, and blood vessel cells were successfully identified in zebrafish ovaries [18]. However, the single-cell report on the ovary of the Asian sea bass (*Lates calcarifer*) only defined germ cells, granulosa cells, and somatic cells [16].

This study identified six cell types in the ovary of Sichuan bream, including germ cells, follicular cells, immune cells, stromal cells, epithelial cells, and endothelial cells, utilizing known typical marker genes. Their respective biological functions were verified through GO enrichment analysis. The consistent identification of germ cells, follicular cells, and immune cells across multiple fish ovarian studies suggests the evolutionary conservation of these cell types, potentially representing fundamental functional components of teleost ovaries. Notably, the follicular cells in fish are homologous to the granulosa cells in mammals, which are typically categorized into an inner layer of granulosa cells and an outer layer of theca cells [44,45,46]. In addition to providing mechanical and nutritional support to oocytes, follicular cells primarily function to produce estrogen, which regulates oocyte growth and development [3,47]. Studies in mammals have shown that estradiol (E_2_) production requires the coordinated interaction between granulosa and theca cells. After low-density lipoprotein (LDL)-cholesterol enters granule cells or follicular membrane cells as the substrate, it produces intermediate products required for E_2_ synthesis through a series of enzymatic reactions under the influence of gonadotropin. These products are then transported from theca cells to granulosa cells via the basal lamina, with the final synthesis of E_2_ predominantly occurring in granulosa cells [48]. Unfortunately, we were unable to further subdivide the follicular cell population due to a lack of specific marker genes. The identification of cell types relies heavily on the specific expression of marker genes, which are relatively conserved across various species. However, the diversity among fish species can result in significant variations in genetic backgrounds [49,50]. Therefore, the development of more species-specific marker genes is essential for advancing and deepening single-cell research.

### 3.2. Genetic Dynamics of Germ Cell Development

The development of the reproductive system is a multifaceted and intricate process influenced by various factors. To gain deeper insights into the regulation of genes governing germ cell development, we extracted and re-clustered the germ cell populations. Three subtypes were identified based on the expression of specific genes at different developmental stages: early oogonia, mitotic oogonia, and oocytes. Reproductive cells at each developmental stage exhibit significant heterogeneity and distinct transcriptional characteristics. Furthermore, by leveraging the advantages of single-cell sequencing technology, we accurately classified each germ cell subtype according to their gene expression profiles at different developmental stages and reconstructed the cell development process through pseudotime trajectory analysis. Interestingly, during the pseudotime developmental trajectory of germ cells, early oogonia exhibit an “additional branch” in the middle and late stages of development, implying their potential to differentiate into other cell types. This observation suggests that early oogonia may possess more stage-specific transcriptional patterns. In studies on early human germ cells, it has been found that there exists a stage of response to retinoic acid (RA) signaling prior to the initiation of the first meiosis in oogonia, referred to as the RA-responsiveness stage [51,52]. In mammals, the transmission of RA signals can promote meiosis in germ cells [53,54]. We observed significant expression of the marker genes *zglp1* and *thrap3*, which are associated with RA signaling in the different types of oogonia [40]. A previous study indicated that follicular and stromal cells in zebrafish ovaries may produce retinoic acid, as these cells express *aldh1a2*, which encodes the enzyme responsible for converting retinaldehyde into retinoic acid [55]. However, due to the limited reports available on fish, we did not define the subtype of RA-responsive oogonia. Nevertheless, the expression of these potential stage-specific genes partially elucidates our experimental results.

Elucidating the typical genetic characteristics and potential functions of different subtypes is crucial for understanding the genetic dynamics of germ cells at various developmental stages. Among these, *pou5f1* is a multifunctional transcription factor expressed in embryonic stem cells, early germ cells, and primordial germ cells [56]. Our findings indicate that *pou5f1* is significantly expressed in early oogonia, alongside several oogonia marker genes such as *prdm10*, *klf4*, *dazl*, *sox4*, *sall4*, *lin28a*, *top2a*, and *dnmt3b*. These genes have been extensively studied across various species, including humans [11], mice [13], yaks [14], and zebrafish [18]. During oogonia development, continuous proliferation through mitosis is essential. This study found that the DNA replication licensing factor Mcms and cell cycle regulatory factors (*ccnb1*, *ccna1*) were highly expressed in mitotic oogonia, aligning with the functional characteristics of cells at this stage. In oocytes, in addition to the typical germ cell marker genes *zar1* and *piwil1*, we observed a substantial upregulation of genes associated with ribosomal proteins (Rps), suggesting an increased demand for robust protein synthesis to support rapid cellular growth during this developmental phase. This finding is consistent with prior research on Asian sea bass [16]. In oviparous animals, substantial quantities of mRNA are transcribed and stored in the maternal cytoplasm during oogenesis, providing essential factors for oocyte growth and the maintenance of cell viability [57]. Consequently, precise regulation of protein accumulation is critical, necessitating a significant accumulation of ribosomes in germ cells to efficiently translate this information. Furthermore, this study investigated the expression patterns of typical germ cell marker genes across different developmental stages. Notably, zona pellucida sperm-binding proteins (*zp3*, *zp4*) and DNA methyltransferase (*dnmt1*) exhibited stable high expression levels at each developmental stage. Similarly, studies of human germ cells have shown that the expression levels of *ZP2*, *ZP3*, and *ZP4* remain stable throughout all stages of development [11]. Our analysis indicates that *zp3*, *zp4*, and *dnmt1* may constitute the fundamental functional components of Sichuan bream germ cells.

Transcription factors (TFs) and their regulatory networks are crucial in determining cellular characteristics. Among the TFs expressed during the early oogonia stage, *SOX17* has been identified as an indispensable factor in the formation of human primordial germ cells [58,59]. The interplay between *SOXs* and *POU5F1* is vital for maintaining the balance between stemness and stability in early oogonia [60]. Specific protein 1 (*Sp1*) is implicated in numerous cellular processes [61]. A recent study has revealed the presence of *Sp1* in both germ cells and the surrounding somatic cells of mice. Experiments involving *Sp1* knockout have demonstrated its critical role in the formation of primordial follicles in mice [62]. Histone deacetylase 2 (*Hdac2*) regulates gene expression by reshaping the cellular chromatin structure [13], suggesting that early oogonia may influence their developmental fate through self-induced epigenetic modifications. Furthermore, the upregulation of *dtl* and *phf8* has been observed; both of these factors have been shown to selectively target several key cell cycle regulatory proteins while maintaining precise control over the cell cycle [63,64]. Among the TFs specific to the mitotic oogonia type, TATA-box binding protein associated factor 1 (*taf1*) has been found to play a central role in regulating the expression of most eukaryotic genes [65], particularly in controlling the transcription of numerous genes related to cell division [66]. Additionally, *rara* has been identified as a key regulator of retinoic acid receptor alpha, which may be closely linked to the initiation process of subsequent meiosis [67].

In the oocyte type, the specific expression of the transcription factor X-box binding protein 1 (*xbp1*) has been detected; this factor is upregulated in response to endoplasmic reticulum stress and is widely recognized for its role in promoting lipogenesis [68,69,70]. We speculate that the upregulation of *xbp1* may be associated with the lipid requirements of oocytes during development, particularly in the vitellogenesis stage, when oocytes require substantial lipid nutrition for yolk accumulation. A recent study highlighted the critical role of EP300-mediated crotonylation in oocyte maturation by activating the EGFR signaling pathway and regulating critical cellular processes, such as proliferation and apoptosis. Subsequent experiments utilizing both in vitro maturation (IVM) models in mice and *EP300* knockout mice further corroborated these findings, thereby positioning EP300 as a pivotal regulator in reproductive health [71]. DNA polymerase epsilon subunit 4 (*pole4*) is a subunit of the DNA polymerase epsilon (Polε) complex, primarily involved in DNA replication and repair [72]. Although direct studies on *pole4* in oocyte development are limited, its known biological functions suggest a potential critical role in oocyte maturation and development. During meiosis, oocytes undergo extensive DNA replication and recombination, and any errors in these processes can lead to chromosomal abnormalities or meiotic arrest [73]. As part of the Polε complex, *pole4* likely ensures accurate DNA replication, thereby maintaining oocyte genome integrity [74]. Furthermore, *pole4* may interact with DNA repair factors such as BRCA1 and ATM to coordinate DNA damage responses essential for oocyte quality and developmental potential [75,76]. Additionally, *pole4* may influence epigenetic modifications during replication [77], ensuring the proper transmission of epigenetic marks necessary for oocyte maturation and early embryonic development.

### 3.3. DEGs’ Function in Follicles Before and After Vitellogenesis

The comparison of ovarian samples at stages II and III revealed a significant reshaping of cellular characteristics in the ovaries due to vitellogenesis. Both germ cells and follicular cells exhibited notable alterations in cell number proportions and DEG counts. As the most essential functional cells in the ovary, germ cells undergo growth throughout the entire ovarian development process, particularly during vitellogenesis, when their cell diameter significantly increases to accommodate nutrient accumulation [78]. This study found that the “insulin signaling pathway” was significantly enriched among the upregulated DEGs in stage III germ cells. Recent studies have underscored the central role of the insulin signaling pathway in regulating oocyte growth and maturation, with a prevailing consensus that the expression of insulin-like growth factors (IGFs) promotes oocyte growth [79,80]. Our findings indicated a general increase in the expression of IGF-binding proteins (IGFBPs) in stage III germ cells, including *igfbp1*, *igf2bp1*, and *igf2bp3*, which have been reported to interact with IGFs and contribute to IGF signaling in vivo [81,82].

The origin of fish yolk substances has long been a topic of debate. Vitellogenesis in oviparous animals can be categorized into autosynthesis and heterosynthesis based on whether the yolk is produced within the oocyte or sourced from other extracellular tissues [83]. Our analysis revealed that the upregulated DEGs in stage III germ cells were significantly enriched in the pathways of “protein processing in endoplasmic reticulum” and “endocytosis”. These pathways correspond to the mechanisms of protein intake following intracellular or extracellular production in oocytes, suggesting that the yolk substances in Sichuan bream may derive from both autosynthesis and heterosynthesis. Furthermore, a substantial number of upregulated DEGs were found to be primarily involved in the “MAPK signaling pathway”, “mTOR signaling pathway”, and “FoxO signaling pathway”. These pathways are centrally located within the KEGG network and are extensively interconnected with various biological processes. Notably, recent studies have indicated that MAPK signaling plays a critical role in lipid metabolism in the ovaries of crustaceans, thereby contributing to vitellogenesis in mud crabs (*Scylla paramamosain*) [84]. Additionally, the mTOR signaling pathway has been identified as a key regulator of protein intake [85]. Vitellogenin (Vtg), the most significant yolk substance, is a large phospholipoglycoprotein, and the coordination among these signaling pathways involved in nutrient metabolism is crucial for the production, intake, and accumulation of Vtg in oocytes.

Follicular cells are the most critical type of somatic cells within the ovary. Research indicates that the development of follicular cells is closely coordinated with the maturation process of oocytes [86,87]. However, the stage-specific transcriptional changes occurring in follicular cells remain poorly understood. This study found that the “Notch signaling pathway” and “Hippo signaling pathway” were significantly enriched in the upregulated differentially expressed genes (DEGs) of stage III ovarian follicular cells. The roles of these pathways in follicular cells have been recently documented, primarily mediating essential signals for reproductive cells that regulate follicular cell proliferation during oogenesis [88,89]. Furthermore, DEGs associated with the “DNA replication” and “cell cycle” pathways also contribute to the proliferation of follicular cells. Once formed, the oocyte is promptly surrounded by follicular cells, relying on them for factors that it cannot synthesize independently [90]. Notably, the upregulated DEGs in stage III follicular cells were significantly enriched in the “pyruvate metabolism” pathway, which is vital for oocyte maturation. Studies have shown that oocytes exhibit lower metabolic efficiency for glucose, whereas follicular cells can utilize glucose and convert it into intermediate products such as pyruvate. These intermediate products are subsequently transferred to oocytes through gap junctions, providing energy substrates necessary for oocyte development and thereby mitigating the nutritional depletion of oocytes during vitellogenesis [91,92]. In addition to coordinating energy metabolism, follicular cells supply oocytes with nutrients, hormones, and steroids through the “protein processing in endoplasmic reticulum” and “protein export” pathways.

During vitellogenesis, one of the primary functions of follicular cells is to synthesize estrogen, which stimulates the production of Vtg [93]. We observed that DEGs in stage III follicular cells were significantly enriched in the “cholesterol metabolism” and “estrogen signaling pathways”. Compared to stage II, the expression of *cyp11a1* and steroidogenic acute regulatory protein (StAR)-related lipid transfer proteins, *stard10* and *stard13*, was significantly upregulated in stage III follicular cells. The transfer of cholesterol from the mitochondrial outer membrane to the inner membrane via StAR represents the first rate-limiting step in estrogen biosynthesis, followed by the conversion of cholesterol into pregnenolone, mediated by cytochrome P450 cholesterol side-chain cleavage enzyme *cyp11a1* [93]. Additionally, we noted a significant upregulation of *cyp11a1* expression in stage III follicular cells, which facilitates the conversion of androstenedione into estradiol through aromatization, marking the final step in steroid hormone biosynthesis.

### 3.4. Cellular Interaction Networks During Vitellogenesis

The ovary is a highly heterogeneous multicellular tissue, and the growth and maturation of oocytes depend significantly on intricate and coordinated intercellular communication [94]. This study established the intercellular communication network of stage III ovaries based on the expression levels of ligand–receptor pairs. The results indicate that interactions among germ cells, as well as between germ cells and somatic cells, are primarily mediated by “cell adhesion molecules”, the “TGF-beta signaling pathway”, the “Wnt signaling pathway”, and “ECM-receptor interactions”. These pathways have been partially documented in mammalian ovarian single-cell studies [15,95]. However, a significant research gap remains in the single-cell analysis of fish ovaries, particularly during the vitellogenesis stage.

The extracellular matrix (ECM) is involved in numerous physiological processes in the ovary, with its core component, fibronectin 1 (*fn1*), demonstrating a high likelihood of communication across most examined cell types. This suggests that the ECM plays a vital role in sustaining ovarian function. Research has shown that the ECM significantly influences folliculogenesis, corpus luteum formation, and ovulation in both mammals and humans [96]. Additionally, *lamb1a*, another critical ligand in this pathway, has been identified as playing a role in the proliferation, migration, adhesion, and development of porcine ovarian granulosa cells [97]. Regarding the TGF-beta signaling pathway, its ligand *gdf9* and receptor *bmpr2* are present in oocytes, consistent with findings in studies on mouse and human ovaries [11,13]. The TGF-beta signaling pathway can be activated through autocrine mechanisms to regulate cellular development. Growth differentiation factor 9 (*gdf9*), a member of the transforming growth factor β (TGF-β) superfamily, is crucial for regulating follicle development. Studies on *Monopterus albus* [98] and *Dicentrachus labrax* [99] have shown that the mRNA expression of *gdf9* significantly increases prior to vitellogenesis, which is essential for the growth of primary oocytes. The development and maturation of the ovary are characterized by structural changes that require the involvement of intercellular adhesion molecules [100]. This study found that germ cells establish tight connections with other cells primarily through *jam2a*, *oclns*, and *nectin3b*. A report on zebrafish indicates that the interaction between junctional adhesion molecules (Jams) facilitates the transmission of Notch signals, which may be essential for the proliferation and functional maintenance of follicular cells [101]. The occludin (Ocln), an integral membrane protein found within tight junctions, was observed to be expressed in both oocytes and follicular cells. We detected Ocln (*oclna* and/or *oclnb*) in both oocytes and follicular cells, which are integral membrane proteins that contribute to the functionality of tight junctions. Some studies have suggested that Ocln may serve as a key regulator of yolk accumulation by promoting the transfer of yolk substances to the oocyte surface through the stimulation of vitellogenin-specific receptor LRs, thereby enhancing the efficiency of vitellogenesis [102,103].

## 4. Materials and Methods

### 4.1. Ethics Statement

The experimental protocols were approved by Southwest University, and the study was conducted following the guidelines set forth by the Institutional Animal Care and Use Committee of Southwest University (IACUC No. Approved: IACUC-20220623-01).

### 4.2. Animal Preparation and Animal Tissue Collection

The experimental fish used in this study were artificially bred Sichuan bream (*Sinibrama taeniatus*), with parent stock sourced from the natural rivers of the Upper Yangtze River. The breeding and daily management of these experimental fish were conducted in accordance with our previous reports [4]. For the single-nucleus RNA sequencing (snRNA-seq) study, we selected ovaries from juvenile Sichuan bream that had developed to 115 days (n = 3) and 165 days (n = 3) post-hatching. At these developmental stages, the ovaries were classified as being in the pre-vitellogenesis stage (stage II) and the vitellogenesis stage (stage III), respectively [4]. The ovaries were dissected and isolated after anesthetizing the experimental fish with tricaine methanesulfonate (MS-222). Following the removal of excess tissue, each ovary sample was washed with phosphate-buffered saline (PBS), cut into fragments smaller than 5 mm^3^, transferred to a 2 mL tissue Dounce homogenizer (Sigma, #D89381SET, St. Louis, MO, USA) containing 2 mL of pre-cooled lysis buffer (0.1% IGEPAL, 10 mM Tris-HCl, 10 mM NaCl, and 3 mM MgCl_2_), and incubated on ice for 5 min. Each sample was homogenized and subsequently passed through a 70 μm cell strainer (Sigma, #CLS431752-50EA) to remove debris. An additional 3 mL of pre-chilled lysis buffer was then added, followed by centrifugation at 4 °C and 500× *g* for 5 min to isolate the nuclei. Next, 5 mL of nucleus suspension buffer (NSB), consisting of PBS, 0.01% BSA, and 0.1% RNase inhibitor (Clontech, cat. no. 2313A, Mountain View, CA, USA), was added to wash the cell nucleus precipitate. Finally, the isolated nuclei were resuspended in 2 mL of NSB, filtered through a 35 μm cell strainer (Corning Falcon, cat. no. 352235, Guangzhou, China), and counted to ensure a final concentration of 1000 nuclei/μL for 10× Genomics sequencing.

### 4.3. snRNA-Seq Library Construction and Sequencing

Using the 10× Genomics Chromium™ system, sequentially labeled gel beads, sample and reagent premixes, and oils were loaded into their respective sample injection channels through the “T-shaped channel” formed by the microfluidic channel network. This process ultimately results in the formation of a single-cell microreaction system, known as Gel Beads in Emulsion (GEMs), which are encapsulated by oil droplets. Following the successful formation of GEMs, complementary DNA (cDNA) was synthesized via independent reverse transcription within each GEM using a PCR apparatus. After conducting a quality inspection, the labeled cDNA was pooled, amplified, and subsequently utilized for library construction. A second quality inspection was performed on the constructed library, which was then sequenced on a NovaSeq 6000 (Illumina, San Diego, CA, USA) with a target of 150 base pair paired-end reads, ensuring that the sequencing data volume was maintained at or above 20,000 reads per cell.

### 4.4. Data Processing and Downstream Analysis

Following the quality control of raw sequencing data using Cell Ranger (version 7.0.0), the sequencing reads were aligned to our unpublished reference genome of *Sinibrama taeniatus* using the STAR software (version 2.7.9a). This process yielded high-quality cell barcodes, gene counts, genome alignment information, and other relevant results from the sample data. Subsequently, Seurat software (version 3.1.0) was employed for further quality control filtering, during which unqualified data, such as doublets, dead cells, and cell fragments, were removed. This removal was based on several criteria, including the number of genes expressed in each cell, the number of unique molecular identifiers (UMIs), mitochondrial gene expression, ribosomal gene expression, and additional metrics, thereby ensuring the reliability of the subsequent analysis results. The raw single-nucleus RNA sequencing data can be accessed from the NCBI Gene Expression Omnibus database (BioProject ID: PRJNA1195649).

Batch effect removal and the integration of multiple sample datasets were performed using the Seurat package (version 4.0.0), which facilitates the integration of data from different samples. Principal component analysis (PCA) was employed to identify the principal components (PCs) for integration into the dataset, with the top 30 PCs selected for subsequent analysis. The dataset was clustered using the FindClusters function in the Seurat package (version 4.0.0), based on the local neighborhood of the cells, with the resolution parameter set to 0.5. The clustering results were then visualized using t-distributed stochastic neighbor embedding (t-SNE). Subsequently, the FindMarkers function in the Seurat package (version 4.0.0) was utilized to identify differentially expressed genes (DEGs) between two distinct samples or clusters, with DEGs exhibiting |log2FC| > 0.25 and *p* < 0.05 considered significantly different. Additionally, Gene Ontology (GO) and Kyoto Encyclopedia of Genes and Genomes (KEGG) functional enrichment analyses were conducted using Bonferroni-corrected *p*-values for the related DEGs, with a threshold of *p* < 0.05 indicating statistical significance.

### 4.5. Pesudotemperal Trajectory Analysis

The Monocle2 package was employed to analyze the gene expression matrix of germ cells. We selected ordered genes to delineate cell progression and utilized DDRTree to reduce the dimensionality to two dimensions. The pseudotime trajectories of the distinct cell types were visualized using the “plot cell trajectory” function. Heatmaps for the highly variable genes and signature genes along the pseudotime were generated using the “plot pseudotime heatmap” function.

### 4.6. Transcriptional Regulation Analysis of Germ Cell Subtypes

After comparing the genome of *Sinibrama taeniatus* with human homologous genes, transcription factors (TFs) in the target group were identified and analyzed using the pySCENIC software (version 0.11.2), based on the classification of germ cell subtypes. The results were visualized using the “RuntSNE” function in the Seurat package (version 4.0.0).

### 4.7. Cell Communication Analysis

Based on the one-to-one homologous genes of humans and *Sinibrama taeniatus*, the “CellChat” Python package (version 1.1.3) was utilized to analyze ovarian intercellular communication. Receptors or ligands expressed in at least 10% of a specific cell type were subsequently examined, with a significance threshold set at *p* < 0.05. The relationships and strengths of the interactions among the cell types were visualized using the “circlize” and “heatmap” R packages (version 4.3.1).

## 5. Conclusions

In summary, a single-cell atlas of the Sichuan bream ovary was constructed by identifying six cell types. Subsequently, the developmental processes of germ cells were reconstructed, potential functional marker genes at each developmental stage were identified, and the complex regulatory effects of transcription factors on germ cells at various developmental stages were analyzed. Additionally, the transcriptional dynamics of germ cells and follicular cells before and after vitellogenesis were compared. Finally, a communication network between germ cells and other somatic cells, as well as among germ cells themselves, was established through cell interaction analysis. This study is the first to apply single-cell transcriptome sequencing technology to investigate the molecular regulatory mechanisms of fish ovaries during vitellogenesis. It provides a novel perspective for the in-depth study of fish reproductive physiology.

## Figures and Tables

**Table 1:** Cell quality assessment and filtering.

Sample	nCount RNA	nFeature RNA	Percent Double Cell	Num Orig	Num Filterd
II-1	13,963	200–4132	8.41%	11,073	9448
II-2	11,337	200–3595	9.64%	11,514	9660
II-3	16,998	200–4512	5.90%	7524	6593
III-1	31,489	200–6869	10.80%	12,665	10,701
III-2	31,489	200–6869	10.80%	12,665	10,701
III-3	17,891	200–5316	8.27%	10,242	8786

nCount RNA: the number of UMI per cell; nFeature RNA: the number of genes detected in each cell; Percent double cell: the percentage of the number of cells judged to be doublets; Num Orig: number of cells before filtration; Num filterd: number of cells after filtration.

## Data Availability

The raw single-nucleus RNA sequencing data can be accessed from the NCBI Gene Expression Omnibus database (BioProject ID: PRJNA1195649).

## Supplementary Materials

The following supporting information can be downloaded at https://www.mdpi.com/article/10.3390/ijms26052265/s1.
