# Supplementary material for: Characterizing the Ovarian Cytogenetic Dynamics of Sichuan Bream (Sinibrama taeniatus) During Vitellogenesis at a Single-Cell Resolution
Source: Int J Mol Sci. 2025 Mar 4;26(5):2265. doi: 10.3390/ijms26052265 (PMC11900179; doi:10.3390/ijms26052265)
Supplement: Supplementary file 1 [file ijms-26-02265-s001.zip › ijms-3430055-supplementary.pdf]

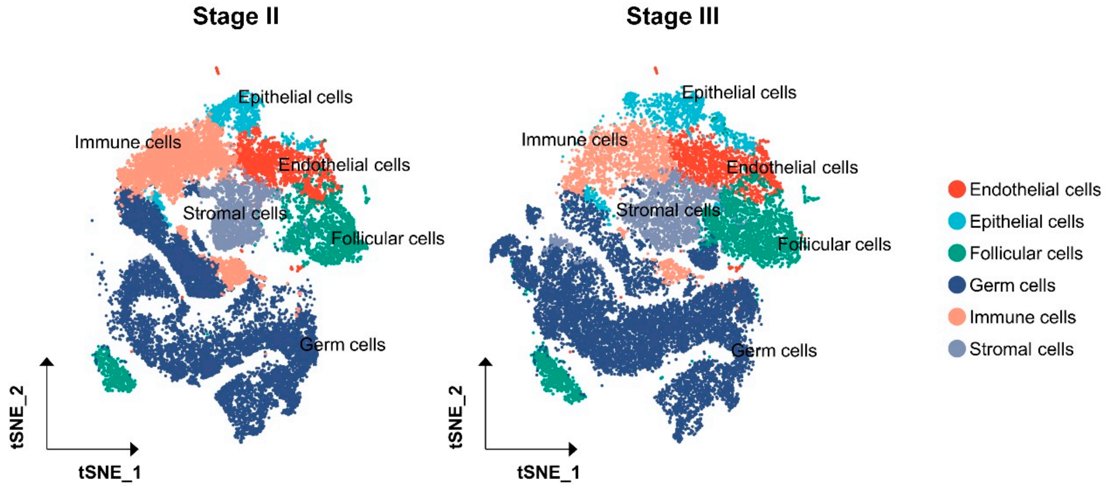

Supplementary Figure S1. t-SNE analysis demonstrating the clustering and identification of ovarian cells at different developmental stages.
